# Dietary Fibre Intake, Adiposity, and Metabolic Disease Risk in Pacific and New Zealand European Women

**DOI:** 10.3390/nu16193399

**Published:** 2024-10-07

**Authors:** Nikki Renall, Benedikt Merz, Jeroen Douwes, Marine Corbin, Joanne Slater, Gerald W. Tannock, Ridvan Firestone, Rozanne Kruger, Lisa Te Morenga

**Affiliations:** 1 School of Sport, Exercise and Nutrition, College of Health, Massey University, Auckland 0632, New Zealandr.kruger@griffith.edu.au (R.K.); 2 Riddet Institute, Centre of Research Excellence, Massey University, Palmerston North 4472, New Zealand; l.temorenga@massey.ac.nz; 3 Research Centre for Māori Health and Development, Massey University, Wellington 6021, New Zealand; 4 Department of Physiology and Biochemistry of Nutrition, Max Rubner-Institut, 76131 Karlsruhe, Germany; benedikt.merz@mri.bund.de; 5 Centre for Public Health Research, Massey University, Wellington 6021, New Zealand; j.douwes@massey.ac.nz (J.D.); m.corbin@massey.ac.nz (M.C.); r.t.firestone@massey.ac.nz (R.F.); 6 Department of Microbiology and Immunology, University of Otago, Dunedin 9016, New Zealand; gerald.tannock@otago.ac.nz; 7 School of Allied Health and Social Work, Griffith University, Gold Coast 4215, Australia

**Keywords:** dietary fiber, adiposity, obesity, metabolic disease, socioeconomic factors

## Abstract

Background/Objectives: To assess associations between dietary fibre intake, adiposity, and odds of metabolic syndrome in Pacific and New Zealand European women. Methods: Pacific (n = 126) and New Zealand European (NZ European; n = 161) women (18–45 years) were recruited based on normal (18–24.9 kg/m^2^) and obese (≥30 kg/m^2^) BMIs. Body fat percentage (BF%), measured using whole body DXA, was subsequently used to stratify participants into low (<35%) or high (≥35%) BF% groups. Habitual dietary intake was calculated using the National Cancer Institute (NCI) method, involving a five-day food record and semi-quantitative food frequency questionnaire. Fasting blood was analysed for glucose and lipid profile. Metabolic syndrome was assessed with a harmonized definition. Results: NZ European women in both the low- and high-BF% groups were older, less socioeconomically deprived, and consumed more dietary fibre (low-BF%: median 23.7 g/day [25–75-percentile, 20.1, 29.9]; high-BF%: 20.9 [19.4, 24.9]) than Pacific women (18.8 [15.6, 22.1]; and 17.8 [15.0, 20.8]; both *p* < 0.001). The main source of fibre was discretionary fast foods for Pacific women and whole grain breads and cereals for NZ European women. A regression analysis controlling for age, socioeconomic deprivation, ethnicity, energy intake, protein, fat, and total carbohydrate intake showed an inverse association between higher fibre intake and BF% (β= −0.47, 95% CI = −0.62, −0.31, *p* < 0.001), and odds of metabolic syndrome (OR = 0.91, 95% CI = 0.84, 0.98, *p* = 0.010) among both Pacific and NZ European women (results shown for both groups combined). Conclusions: Low dietary fibre intake was associated with increased metabolic disease risk. Pacific women had lower fibre intakes than NZ European women.

## 1. Introduction

Aotearoa New Zealand (NZ) has one of the highest rates of obesity in the world, which continues to rise [1]. In the most recent national health survey (2022/23), one in three adults over the age of 15 had a body mass index (BMI) ≥ 30 kg/m^2^ [1]. Obesity rates are higher among Māori (48%) and Pacific peoples (67%) compared to NZ Europeans (32%) [1]. This increased prevalence is also associated with a greater risk of non-communicable diseases (NCDs), such as type 2 diabetes mellitus (T2DM) and cardiometabolic diseases [2,3,4]. This is partially explained by differences in dietary intakes, as well as socioeconomic circumstances [1,4]. Diet is a key modifiable risk factor for developing NCDs [5], yet a healthy diet is not easily accessible or affordable for all in Aotearoa NZ. People living in neighbourhoods characterised by higher socioeconomic deprivation are twice as likely to be obese compared to those living in wealthier areas, and Māori and Pacific people are more likely to live in these neighbourhoods than NZ Europeans [1,4].

Regularly consuming a healthy balanced diet rich in plant-based dietary fibre from fruit, vegetables, whole grains, and legumes is associated with living a longer, healthier life and a reduced risk of developing chronic diseases like heart disease, T2DM, and some cancers [5,6]. Accordingly, since the 1990s food-based dietary guidelines have consistently promoted higher fibre intakes through daily servings of fruit, vegetables, and grains to support overall health and prevent NCDs [7,8]. However, despite the health benefits, dietary fibre intakes in Aotearoa NZ have historically fallen short of the recommended intakes (i.e., ≥25 g/day for women and ≥30 g/day for men [9]). For instance, in the last NZ national nutrition survey (2008/09), the median fibre intakes were 17.5 g (10th–90th centiles: 11.9–24.5 g) for women and 22.1 g (10th–90th centiles: 15–31.5 g) for males [10]. Without up-to-date national nutritional data, the current fibre intakes of the population are relatively unknown; however, recent data from national health surveys (2018/19 and 2019/20) suggest it has not improved much. These surveys showed that only 33% of adults met the recommended number of servings of fruit and vegetables each day, with women and individuals living in wealthier areas more likely to meet the recommendations than men and those living in more deprived neighbourhoods, respectively [11].

Fibre intake may, at least partially, explain some of the differences in obesity prevalence between Pacific and NZ European peoples. Moreover, obesity is particularly concerning for women of childbearing age as excess adiposity increases metabolic disease risk and the likelihood of obesity in the next generation [12]. The aims of this study were to: (1) compare habitual dietary fibre intakes in healthy NZ European and Pacific women aged 18–45 years with different metabolic disease risk and body fat profiles (normal weight and obese); and (2) to explore associations between dietary fibre intake, adiposity, and risk of metabolic syndrome in these groups.

This article is a revised and expanded version of an abstract entitled Dietary fibre intake, adiposity, and metabolic disease risk in Pacific and New Zealand European women, which was presented at the 57th Nutrition Society of New Zealand and 47th Nutrition Society of Australia Joint Annual Scientific Meeting, Nutrition & Wellbeing in Oceania at Massey University, Auckland, New Zealand, 1 December 2023 [13].

## 2. Materials and Methods

### 2.1. Study Design

The participants were part of the cross-sectional predictors linking obesity and gut microbiome (PROMISE) study, which was conducted between July 2016 and September 2017, and aimed to characterise the gut microbiome and related parameters in two population groups with different metabolic disease risks (Pacific and NZ European women) and different body weight profiles (normal weight and obese) according to the WHO classification. Participants were recruited based on self-reported BMI so that half in each ethnic group had either a normal BMI (18–24.9 kg/m^2^) or were obese (BMI ≥ 30 kg/m^2^). As individuals with the same BMI can have different body fat profiles and metabolic disease risks [14,15], participants were subsequently stratified into two body fat percentage (BF%) groups: low-BF% (<35%) and high-BF% (≥35%) for analyses based on international BF% indicators of obesity [14,16,17].

Women were eligible to participate in the study if they were post-menarche and premenopausal (aged 18–45 years), residents in Auckland (NZ), and free from any chronic illness or disease. Details of the PROMISE study procedures and recruitment strategies are reported elsewhere [18]. Data were collected at two clinic visits, 11–14 days apart and at home between visits. Ethical approval for the PROMISE study was provided by the Southern Health Disability Ethics Committee (16/STH/32) and it was registered at anzctr.org.au (ACTRN12618000432213). The study was conducted according to the guidelines of the Declaration of Helsinki, and all participants gave written informed consent prior to participation.

### 2.2. Assessment of Demographic, Anthropometric and Metabolic Risk Factors

Standardised face-to-face interviews captured demographic information. The NZ Deprivation Index (NZDep2013), an area-based measure of socioeconomic deprivation, was used to assign a socioeconomic deprivation score ranging from one “least deprived” to ten “most deprived” [19]. Blood pressure was measured after a 10-min (sitting) period with a digital blood pressure monitor (Omron HEM-907, Omron Healthcare Inc., Kyoto, Japan). A whole-body scan was conducted with Dual-energy X-ray Absorptiometry (DXA) (Hologic QDR Discovery A, Hologic Inc, Bedford, MA, USA with APEX V. 3.2 software) to assess total body fat percentage (BF%) and visceral fat mass (VAT%). Blood from participants was collected following an overnight fast. Blood chemistry assays (glucose and lipid profile) were conducted using standard diagnostic methods (see Appendix A). The presence of metabolic syndrome was assessed using the harmonized definition, i.e., having 3 out of the 5 following risk factors: elevated waist circumference ≥ 80 cm [20], triglycerides ≥ 1.7 mmol/L, systolic ≥ 130 and/or diastolic blood pressure ≥ 85 mm Hg, fasting blood glucose ≥ 100 mg/dL, and reduced HDL cholesterol < 1.3 mmol/L [21].

### 2.3. Dietary Assessment

Participants completed a five-day, non-consecutive, estimated food record (5DFR) at home between study visits. During study visit two, the 5DFR was reviewed in a face-to-face interview with a NZ registered dietitian, and participants completed an online validated 220-item semi-quantitative NZ Women’s Food Frequency Questionnaire (NZWFFQ) [22] regarding the past thirty day’s food intake. Energy, micro- and macronutrient analyses of the NZWFFQ and 5DFR were completed using FoodWorks9 (Xyris Software (Australia) Pty Ltd., Brisbane, Queensland, Australia) and based on NZ’s food composition database FOODFiles 2016 (Plant & Food Research, NZ). The average daily intake of 36 nutrients (in standard units/day) consumed over one month was calculated for each participant using the validated NCI method [23,24] (see Appendix A). To explore the main food sources contributing to total dietary fibre intake, foods from the 5DFR were allocated to 29 food groups based on similar nutritional composition and characteristics of food groups used in previous studies (Supplementary Table S1) [25].

### 2.4. Statistical Analyses

SAS Enterprise Guide version 7.1 (SAS Institute, Cary, NC, USA) was used for all analyses. The normality of data was assessed using Kolmogorov–Smirnov tests and histograms, and medians [25th, 75th] were used to present all non-normally distributed data. Mann–Whitney tests were used to test for differences between groups. Fasting insulin was logarithmically (ln) transformed to normalise the distributions prior to regression analyses.

Multiple linear regression was used to assess associations between habitual dietary fibre intake and body composition and metabolic markers. Logistic regression was used to assess the association between habitual dietary fibre intake and metabolic syndrome. Analyses were adjusted for deprivation (NZDep2013; see above), age, energy intake, total carbohydrate, fat, and protein intake as potential confounders. Analyses were conducted separately for NZ European and Pacific participants, as well as for both groups combined while adjusting for ethnicity. Further adjustments for both BF% groups were conducted to assess the independent association of dietary fibre intake and metabolic markers. Regression coefficients (β) obtained from log-transformed data represent relative differences and were, therefore, expressed as a ratio calculated by exponentiating the regression coefficient as follows: eβ. The associations between dietary fibre intake and body composition and risk of metabolic syndrome were expressed per 1 g of dietary fibre. Collinearity between variables included in the model was assessed by computing the tolerance and the variance inflation factor (VIF); no collinearity was detected. *p*-values < 0.05 were considered statistically significant.

## 3. Results

### 3.1. Characteristics of the Study Participants

**Figure 1:**
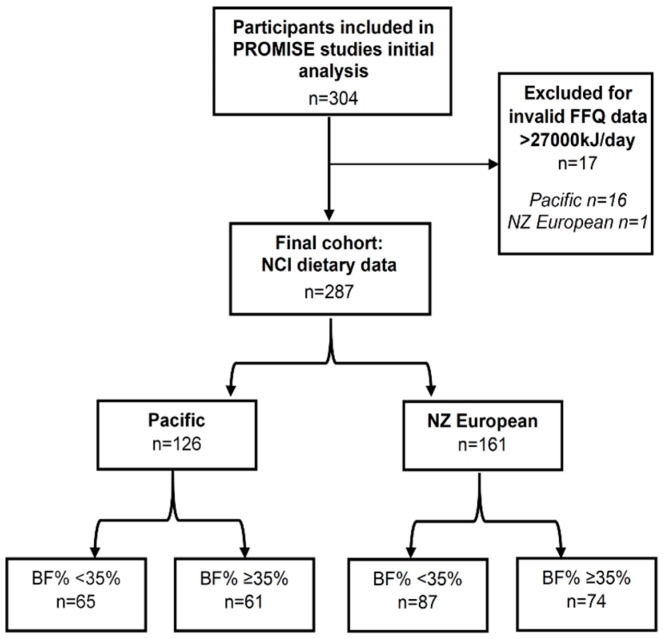
Flowchart of participants included in the current analysis.

A total of 351 women were eligible with 304 completing all aspects of the PROMISE study. Seventeen women were excluded from the analyses based on a reported dietary energy intake in the NZWFFQ of >27,000 kJ/day (Figure 1), leaving 287 participants: 126 (44%) Pacific with a median age of 23 years [25–75-percentile, 20, 29] and 161 (56%) NZ European women with a median age of 32 [25,26] years.

The NZ European women were older, had lower NZDep2013 scores and HbA1c, and higher HDL-C than the Pacific women (Table 1). Among the Pacific women, there was no difference in age or NZDep2013 scores between the low- and high-BF% groups. In contrast, the NZ European women in the high-BF% group were older and had higher NZDep2013 scores than the NZ European women in the low-BF% group. Both Pacific and NZ European women in the high-BF% groups had higher HbA1cs, fasting insulin and glucose, and lower HDL-Cs than the Pacific and NZ European women in the low-BF% groups, respectively.

### 3.2. Dietary Fibre Intake

The NZ European women had a significantly higher median intake of dietary fibre compared to the Pacific women: intakes ranged from 10.7 to 45.9 g/day for the NZ European women and 8.8 to 39.9 g/day for the Pacific women. The NZ European women also consumed a higher percentage of their total daily energy intake from dietary fibre compared to the Pacific women. There was no significant difference in dietary fibre intake between the Pacific women in the low- and high-BF% groups. However, on average, the NZ European women in the low-BF% group consumed more dietary fibre than the NZ European women in the high-BF% group. (Table 1).

#### Nutrient Intake

There was no difference in total fat intake between the Pacific and NZ European women. However, the NZ European women consumed a higher percentage of their total energy intake from PUFA, including less starch, and a lower percentage of their total energy intake from carbohydrates compared to the Pacific women. Additionally, the NZ European women in the low-BF% group consumed less sugar than the Pacific women in the low-BF% group. However, there was no difference in sugar intake between body fat groups within ethnic groups (Table 1).

### 3.3. Association between Dietary Fibre Intake, Metabolic Risk Factors and Metabolic Syndrome

For both the Pacific and NZ European women, their habitual dietary fibre intake was inversely associated with measures of adiposity (weight, BMI, WC, VAT% and BF%, Table 2). Further adjustment for time spent in moderate to vigorous physical activity did not significantly alter the results (Supplementary Tables S2–S4). Habitual dietary fibre intake was associated with a lower likelihood of metabolic syndrome. In particular, there was a 9% lower odds (OR: 0.91 [95% CI: 0.84, 0.98] *p* = 0.010) of metabolic syndrome for every 1 g increase of dietary fibre consumed when analyses were combined for the Pacific and NZ European women. Analyses stratified by ethnicity showed similar ORs, but it did not reach statistical significance for the Pacific women ((OR: 0.90 [95% CI: 0.82, 0.98] *p* = 0.020) in European vs. (OR: 0.93 [95% CI: 0.81, 1.05] *p* = 0.238 in Pacific women) Table 2).

### 3.4. Main Food Sources of Dietary Fibre

**Figure 2:**
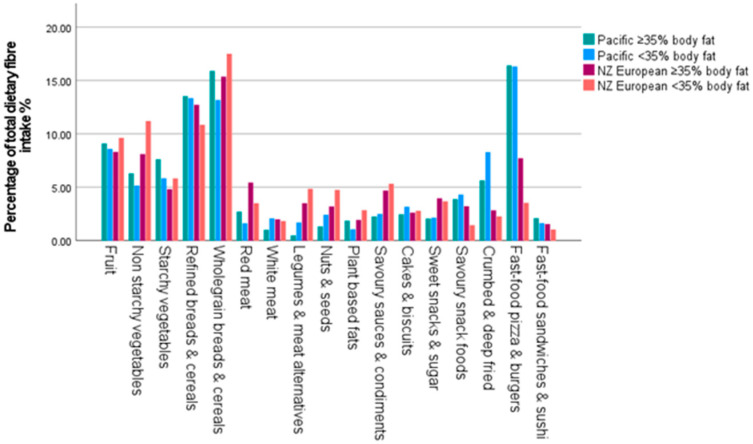
The main food sources contributing to total dietary fibre intake.

The main food sources of dietary fibre differed between groups are shown in Figure 2. Fast food pizza and burgers, whole grain breads and cereals, and refined breads and cereals were the main sources of dietary fibre for Pacific women in both BF% groups; whereas, the NZ European women consumed a higher proportion of their total fibre intake from whole grain and refined breads and cereals, fruit, and non-starchy vegetables (Figure 2).

## 4. Discussion

This study assessed the extent to which differences in adiposity and odds of metabolic syndrome in Pacific versus NZ European women could be explained by dietary fibre intakes. Our study showed that fibre intake is associated with lower adiposity (i.e., weight, BMI, WC, VAT%, and BF%) and metabolic syndrome, as has been demonstrated previously [27,28]. Our study also showed that fibre intakes were low across the whole group of women, for example, 89% of Pacific and 65% of NZ European women did not meet the recommendations (≥25 g/day [9]), and the main sources of fibre intake were discretionary foods for Pacific women suggesting lower overall diet quality. Lower fibre intakes were associated with higher odds of metabolic syndrome and, thus, a higher risk of developing T2DM. However, in analyses stratified by ethnicity, this association was not statistically significant among Pacific women. This is likely to be a consequence of their lower overall fibre intake compared to NZ European women. On average, Pacific women were younger and more likely to live in areas of higher socio-economic deprivation, both factors associated with lower fibre intakes across the whole group.

The association between higher fibre intake and lower risk of nutrition-related NCDs and their metabolic risk factors is well established [5,28]. These benefits have been attributed to the physiochemical and functional properties of dietary fibre [29] and more recently associated with gut microbiota composition and function [30,31,32]. Higher dietary fibre intake may reduce the risk of weight gain by lowering the energy density of the diet, or by increasing faecal bulk that would slow gastric emptying and the rate of nutrient absorption, which, in turn, can influence the production of gastrointestinal hormones (e.g., glucagon-like peptide-1 (GLP-1) and peptide YY), that regulate appetite and insulin secretion [29]. In our study, higher fibre intake was inversely associated with multiple measures of adiposity in both Pacific and NZ European women, including lower odds of metabolic syndrome.

Regularly consuming fibre-rich whole foods such as fruit, vegetables, and whole grains is protective against developing metabolic syndrome and associated NCDs [5,33,34]. Although we did not see a significant association between fibre intake and risk of metabolic syndrome for Pacific women, we did observe statistically significant associations with multiple measures of adiposity. Our findings indicate that increasing fibre intake is still an important health promotion goal for preventing diabetes in Pacific peoples. Increasing dietary fibre intake is challenging as affordable staples tend to be relatively lower in fibre [35,36]. Although we did not measure income directly in our study, we know that Pacific women were more likely to live in areas of high socioeconomic deprivation which is an indicator of low income [4,19]. Further, low-income neighbourhoods also tend to have relatively high and greater concentrations of stores selling fast foods and exposure to the marketing of unhealthy foods [37]. Consequently, those living in high-deprivation neighbourhoods are less likely to meet the recommended number of daily servings of fruit and vegetables [26,38] or guidelines for fibre intake [39], and people living near fast food venues eat more fast food [38]. This may explain, at least in part, that Pacific women who are overrepresented in the higher deprivation groups have lower fibre intakes and that discretionary fast foods were their main source of fibre. Since the 1990′s, dietary guidelines have consistently recommended higher fibre intakes through daily servings of fruit, vegetables, and whole grains to prevent chronic diseases and improve health outcomes [7,8,9]. However, despite continued public health campaigns promoting higher fibre intake [7], our study demonstrates that fibre intake remains low for both Pacific and NZ European women, which is likely contributing to the higher prevalence of obesity and associated NCDs in Aotearoa NZ [1,4]. Public health messaging promoting adequate intakes [7] is clearly not optimally effective, particularly among Pacific people, and affordability (or lack thereof) along with proximity to unhealthy food outlets may indeed be influential factors [37,40].

The design of this cross-sectional study has some limitations. Self-reported dietary data are prone to misreporting but provide important insight into the complexities of what and how individuals are eating that no other set of biomarkers is currently able to provide [41]. Due to the cross-sectional study design, temporal relationships cannot be assessed and causality is not inferred. However, there are several strengths of this study. Extensive methodological approaches to reduce dietary data misreporting were applied successfully [42,43]. For example, the dietary data presented were based on current intake (5DFR) and the inclusion of the NZWFFQ information in the NCI method, which specifically addresses the intra-individual day-to-day variation inherent in dietary data [43], improved intake estimation [24]. In addition, models were adjusted for available participant information, which could influence dietary intake (e.g., other nutrients, deprivation level, age) [43]. Moreover, multiple body composition measurements and metabolic risk factors were assessed, which enabled the opportunity to explore their association with aspects of habitual diet [14,15].

## 5. Conclusions

Our study has shown that low fibre intake was associated with increased metabolic disease risk, and that fibre intake remains low among Pacific and NZ European women. Education and individual actions alone are not sufficient to tackle the costly nutrition-related NCD epidemics. There is an urgent need for policy efforts to target increasing dietary fibre intake in the population in general, and specifically in our low-income and Pacific communities, by ensuring equitable access to nutritious and affordable foods.

## Figures and Tables

**Table 1:** Characteristics of Pacific and NZ European women stratified by BF% groups.

	Pacific		NZ European	
	Low-BF% n = 65	High-BF% n = 61	Low-BF% n = 87	High-BF% n = 74
Age (y)	23 [20, 29]	23 [21, 29]	29 [24, 36] †	35 [28, 40] *†
NZDep2013 ^a^	7 [5, 9]	8 [7, 9]	3 [2, 6] †	5 [3, 6] *†
Body composition				
Weight (kg)	72.4 [67.3, 79.1]	97.0 [87.4, 109.9] *	62.4 [58.1, 66.6] †	94.1 [86.8, 101.7] *
BMI (kg/m^2^)	25.0 [23.6, 27.6]	33.8 [31.1, 39.9] *	22.5 [20.9, 23.5] †	33.5 [31.7, 36.3] *
Waist circumference (cm)	78.1 [75.1, 84.6]	97.0 [89.0, 108.3] *	73.1 [69.5, 75.8] †	97.0 [91.9, 102.8] *
Body fat (%)	29.6 [27.9, 32.3]	39.5 [36.6, 42.4] *	28.0 [24.2, 31.9] †	40.3 [38.7, 44.2] *†
Visceral fat (%)	26.8 [23.1, 31.4]	40.3 [35.6, 43.3] *	21.5 [16.8, 27.3] †	39.7 [35.7, 44.0] *
Metabolic Syndrome (n [%])	5 (8%)	23 (38%)	0 (0%)	29 (39%)
Blood pressure				
Systolic (mmHg)	113 [106, 119]	117 [111, 128] *	113 [105, 119]	120 [111, 128] *
Diastolic (mmHg)	71 [65, 74]	77 [71, 84] *	69 [66, 76]	80 [74, 85] *
Metabolic markers ^b^				
TC (mmol/L)	4.5 [4.1, 5.1]	4.6 [4.2, 5.1]	4.9 [4.3, 5.4] †	5.2 [4.7, 6.1] *†
HDL-C (mmol/L)	1.5 [1.3, 1.8]	1.3 [1.2, 1.6] *	1.8 [1.6, 2.0] †	1.4 [1.3, 1.7] *†
LDL-C (mmol/L)	2.8 [2.4, 3.2]	3.0 [2.5, 3.3]	2.8 [2.4, 3.4]	3.4 [2.7, 4.1] *†
TAG (mmol/L)	0.8 [0.7, 1.1]	1.0 [0.9, 1.5] *	0.7 [0.6, 0.9]	1.1 [0.8, 1.5] *
HbA1c (mmol/L)	32.1 [30.5, 33.8]	34.8 [32.3, 36.7] *	30.6 [29.0, 31.9] †	31.0 [29.8, 33.3] *†
Fasting Glucose (mmol/L)	5.3 [5.0, 5.5]	5.4 [5.1, 5.9] *	5.1 [4.9, 5.3] †	5.5 [5.1, 5.7] *
Fasting Insulin (uU/mL)	11.2 [7.9, 16.0]	21.4 [13.1, 31.9] *	7.1 [5.2, 8.7] †	12.6 [10.0, 17.9] *†
Nutrient intake				
Energy (kJ/day)	8749 [8405, 8986]	8555 [8045, 8894]	8307 [8033, 8660] †	8634 [8188, 8858] *
Protein (E %/day)	15.1 [13.5, 17.2]	15.9 [14.0, 18.4]	16.7 [15.4, 18.1] †	16.9 [15.2, 18.1]
Total fat (E %/day)	39.2 [33.7, 45.1]	39.1 [31.5, 44.0]	41.1 [35.7, 47.1]	39.3 [34.5, 45.9]
SFA (E %/day)	14.9 [12.8, 17.4]	15.5 [12.8, 16.9]	14.8 [12.4, 17.4]	15.1 [13.8, 17.8]
PUFA (E %/day)	5.3 [4.1, 6.0]	4.7 [3.9, 5.8]	6.0 [5.0, 7.0] †	5.3 [4.3, 6.1] *†
MUFA (E %/day)	14.9 [12.7, 16.4]	14.3 [12.3, 17.0]	15.6 [13.2, 17.7]	14.5 [12.4, 17.2]
CHO (E %/day)	40.6 [34.1, 47.5]	42.7 [33.8, 45.6]	35.5 [30.4, 40.3] †	35.8 [31.5, 40.5] †
Sugar (g/day) ^c^	82.5 [74.0, 102.7]	78.9 [66.1, 96.2]	80.2 [66.0, 89.5] †	79.8 [68.8, 95.8]
Starch (g/day)	125.7 [105.1, 145.2]	128.1 [109.1, 146.3]	102.5 [82.0, 119.0] †	104.7 [84.9, 119.8] †
Dietary Fibre (g/day)	18.8 [15.6, 22.1]	17.8 [15.0, 20.8]	23.7 [20.1, 29.9] †	20.9 [19.4, 24.9] *†
Dietary Fibre (g/MJ/day)	2.1 [1.8, 2.5]	2.1 [1.8, 2.4]	2.9 [2.5, 3.5] †	2.5 [2.2, 3.0] *†

Values are reported as median [25th, 75th percentiles]. BF%, Total Body Fat Percentage; Low-BF%, BF% < 35%; High-BF%: BF% ≥35%; Metabolic syndrome n(%) assessed with Harmonised definition [21]; SFA = Saturated fat, PUFA = Polyunsaturated fat, MUFA = Monounsaturated fat, CHO = total carbohydrate. ^a^ Pacific women (n = 2) and ^a^ NZ European woman (n = 1), and ^b^ Pacific woman (n = 1) have not been included in analyses due to missing data. ^c^ Includes all natural/free and added sugars. * Statistically significant difference (*p* < 0.05) between body fat groups within ethnic groups. † Statistically significant difference (*p* < 0.05) between ethnic groups within the body fat group.

**Table 2:** Association between dietary fibre intake, body composition, metabolic markers, and metabolic syndrome.

Variable	All Participants ^a^ β (95% CI) n = 284	*p* Value	Pacific β (95% CI) n = 124	*p* Value	NZ European β (95% CI) n = 160	*p* Value
Body composition						
Weight (kg)	−1.10 [−1.53, −0.66]	*p* < 0.001	−1.61 [−2.59, −0.63]	*p* = 0.014	−1.06 [−1.53, −0.59]	*p* < 0.001
BMI (kg/m^2^)	−0.38 [−0.53, −0.24]	*p* < 0.001	−0.53 [−0.86, −0.21]	*p* = 0.017	−0.38 [−0.53, −0.22]	*p* < 0.001
Waist Circumference	−0.80 [−1.11, −0.49]	*p* < 0.001	−1.01 [−1.69, −0.33]	*p* = 0.004	−0.80 [−1.16, −0.45]	*p* < 0.001
Total body fat %	−0.47 [−0.62, −0.31]	*p* < 0.001	−0.48 [−0.77, −0.19]	*p* = 0.016	−0.48 [−0.68, −0.28]	*p* < 0.001
Visceral fat %	−0.61 [−0.82, −0.40]	*p* < 0.001	−0.59 [−1.01, −0.18]	*p* = 0.006	−0.64 [−0.90, −0.37]	*p* < 0.001
Metabolic markers ^b,c^						
TC (mmol/L)	−0.04 [−0.06, −0.01]	*p* = 0.001	−0.02 [−0.05, 0.02]	*p* = 0.328	−0.04 [−0.06, −0.01]	*p* = 0.016
HDL-C (mmol/L)	0.0004 [−0.01, 0.01]	*p* = 0.910	0.003 [−0.01, 0.02]	*p* = 0.700	0.001 [−0.01, 0.01]	*p* = 0.897
LDL-C (mmol/L)	−0.03 [−0.05, −0.01]	*p* = 0.002	−0.01 [−0.05, 0.02]	*p* = 0.521	−0.04 [−0.06, −0.01]	*p* = 0.015
TAG (mmol/L)	−0.01 [−0.02, 0.01]	*p* = 0.304	−0.01 [−0.04, 0.01]	*p* = 0.333	−0.04 [−0.02, 0.01]	*p* = 0.511
HbA1c (mmol/L) ^d^	−0.005 [−0.07, 0.06]	*p* = 0.882	−0.05 [−0.21, 0.10]	*p* = 0.488	−0.01 [−0.08, 0.06]	*p* = 0.829
Fasting Glucose (mmol/L)	−0.02 [−0.02, 0.0005]	*p* = 0.061	−0.01 [−0.04, 0.01]	*p* = 0.370	−0.01 [−0.02, 0.002]	*p* = 0.125
Fasting Insulin ^e^	0.99 [0.98, 1.00]	*p* = 0.101	0.97 [0.94, 1.00]	*p* = 0.046	0.99 [0.98, 1.01]	*p* = 0.226
Metabolic syndrome (OR) ^f^	0.91 [0.84, 0.98]	*p* = 0.010	0.93 [0.81, 1.05]	*p* = 0.238	0.90 [0.82, 0.98]	*p* = 0.020

All models adjusted for age, NZDep2013, energy, protein, total fat, and carbohydrate intake. ^a^ further adjusted for ethnicity; ^b^ further adjusted for body fat% groups. ^c^ Pacific woman (n = 1) and ^d^ Pacific women (n = 3) not included in analyses due to missing data. ^e^ data has been log-transformed (ln) and is presented as a ratio (eβ). ^f^ OR: Odds ratio. Metabolic syndrome assessed with a Harmonised definition [21]. Regression coefficients represent the change in the outcome per 1 g of change in dietary fibre intake.

## Data Availability

The data presented in this study are available on reasonable request from the corresponding author due to privacy and ethical restrictions.

## Supplementary Materials

The following supporting information can be downloaded at: https://www.mdpi.com/article/10.3390/nu16193399/s1, Table S1: Food Groups; Table S2: Associations between dietary fibre intake, body composition and metabolic markers for Pacific and NZ European women; Table S3: Associations between dietary fibre intake, body composition and metabolic markers for Pacific women; Table S4: Associations between dietary fibre intake, body composition and metabolic markers for NZ European women.

## Appendix A

### Appendix A.1. Methods

#### Blood Analyses

Participants attended a clinic between 7:30 am and 9:00 am following an overnight fast of at least 10 h. Blood was collected into EDTA and silicone-coated (with clot activator) vacutainer tubes, to obtain plasma and serum, respectively. Immediately following blood collection, an aliquot of EDTA whole blood was frozen at −80 °C for HbA1c analysis, and serum tubes were kept at room temperature for 30 min for clot formation. The remaining samples were kept at 4 °C, and within an hour of collection, centrifuged at 3500 rpm for 15 min at 4 °C, and the plasma and serum samples were frozen at −80 °C for later analyses.

Serum levels of glucose (enzymatic UV method), total cholesterol (TC), HDL-C, and LDL-C (enzymatic colourimetric method) were measured using a Hitachi c311 autoanalyser (Hitachi High Technologies Corporation, Tokyo, Japan) with Roche Diagnostic reagents (Mannheim, Germany). Whole-blood HbA1c levels were measured by turbidimetric inhibition immunoassay (Roche Diagnostic, Mannheim, Germany) on a Hitachi c311 autoanalyser (Hitachi High Technologies Corporation, Tokyo, Japan). The average inter-assay coefficient of variation for all the above analyses was below 1.5%. Serum insulin was measured using the electrochemiluminescence immunoassay method (Roche Diagnostics, Mannheim, Germany) on the Cobas e411 analyser (Hitachi High Technologies Corporation, Tokyo, Japan). The inter-assay coefficient of variation was 0.5%.

### Appendix A.2. Power Calculation

The power calculation was originally determined for the whole PROMISE study and based on metagenome-based measures of gene richness (gene counts) of faecal microbial communities which have categorised individuals into clusters of high or low gene count [18]. In the current analyses, assuming a standard deviation of 2 g/MJ/day for dietary fibre intake and a two-sided significance level of 0.05, the sample size of 287 women (65 Pacific low-BF%, 61 Pacific high-BF%, 87 NZ European low-BF%, 74 NZ European low-BF%) would allow detecting differences in means of 1 g/MJ/day and 0.9 g/MJ/day between low-BF% and high-BF% women among Pacific and NZ European women with 80% power, respectively.

### Appendix A.3. Dietary Assessment

All individual reported energy intakes underwent a plausibility assessment by a NZ registered dietitian, with individual daily intake values between 2100 kJ/day and 27,000 kJ/day being considered plausible for valid completion of the 5DFR and NZWFFQ as they are reflective of the food consumed by the population [44,45]. NZ’s food composition database FOODFiles 2016 used the AOAC Prosky method to measure dietary fibre.

### Appendix A.4. Habitual Dietary Intake

The validated NCI method uses a two-part modelling approach to estimate the probability of consumption of a specific nutrient per day and the amount consumed. The NCI method considers the effect of covariates which could influence the probability of consumption (e.g., seasonality) or the amount consumed (e.g., age). For each individual, habitual intake is then defined as the product of the probability of consumption multiplied by the consumed amount (Habitual intake = Probability × Amount) [23,24]. Estimation of a dietary component consumed daily (e.g., energy) requires a one-part model as there is no need to model the probability of consumption (it is assumed to be one). In the current analyses, the 5DFR was used as the primary dietary data and age, ethnicity, BMI, season, NZWFFQ information (in standard units/day), and weekday/weekend information (weekday = Monday–Thursday, weekend = Friday–Sunday) as covariates. If a participant reported never consuming a food group or nutrient in both the NZWFFQ and 5DFR they were considered a “true non-consumer” and were assigned 0 g/day for the food group or nutrient, respectively.

### Appendix A.5. Physical Activity

Physical activity was assessed with a triaxial w-GT3X accelerometer (Actigraph, Pensacola, FL, USA) and the epochs were categorised into time (min/day) spent in various levels of physical activity (counts/min, (cpm)). Detailed methodology is described elsewhere [46]. In this study, a composite measure of moderate and vigorous-intensity physical activity (MVPA; >2020 cpm) was used as current physical activity guidelines recommend ≥ 30 min of moderate (or higher) intensity activity ≥ 5 days of the week [7].
