# Supplementary material for: Dietary Fibre Intake, Adiposity, and Metabolic Disease Risk in Pacific and New Zealand European Women
Source: Nutrients. 2024 Oct 7;16(19):3399. doi: 10.3390/nu16193399 (PMC11479009; doi:10.3390/nu16193399)
Supplement: Supplementary file 1 [file nutrients-16-03399-s001.zip › nutrients-3218776-supplementary.pdf]

Supplementary tables

Supplementary description

Supplementary Table S1: List of food groups used within the current analysis from the 5DFR that were allocated to 29 food groups based on similar nutritional composition and characteristics of food groups used in previous studies.

Supplementary Table S2: Results from additional multivariate linear regression models exploring associations between dietary fibre intake with body composition and metabolic markers for Pacific and NZ European women.

Supplementary Table S3: Results from stratified analysis of multivariate linear regression models exploring associations between dietary fibre intake with body composition and metabolic markers for Pacific women.

Supplementary Table S4: Results from stratified analysis of multivariate linear regression models exploring associations between dietary fibre intake with body composition and metabolic markers for NZ European women.

Supplementary Table S1. Food groups

	Food Group	Food Items Included
1	**Dairy products**	Full and fat milk (including lactose free), dairy yoghurt
2	**Milk alternatives**	Soy, almond, rice, oat, coconut varieties
3	**Cheese**	High and low-fat cheese
4	**Sweetened milk products**	Flavoured milk, fermented or evaporated milk, breakfast drinks, yakult fermented milk drink, hot chocolates, milk based smoothies and drinks from cafés
5	**Fruit**	All fresh, canned, frozen, or dried fruit
6	**Non starchy vegetables**	Tomatoes, green and other non-starchy vegetables (onion, capsicum, frozen mixed vegetables)
7	**Starchy vegetables**	Potatoes, kumara, yam, parsnip, turnip, taro, green banana, sweet corn, breadfruit, cassava, green banana, carrots, pumpkin, butternut squash
8	**Refined breads and cereals**	White bread (including gluten free), crumpets, scones, savoury muffin, plain croissant, pancakes, waffles, iced bun, crackers, refined grains, white rice, white pasta, noodles (instant, egg, rice), canned spaghetti, cous cous, refined grains mixed dishes (macaroni and cheese), refined cereals: cornflakes, rice bubbles sweet and chocolate-based cereal
9	**Wholegrain breads and cereals**	Wholegrain breads (High fibre, wholemeal, wholegrain, including gluten free options) grains (quinoa, buckwheat, bulgur wheat, brown rice, wholemeal pasta, wholegrain gluten free pasta e.g., brown rice), porridge, rolled oats, oat bran, oat sachets, Weetbix, bran cereals, all muesli and granola
10	**Red meat**	Beef, lamb, venison, mince, patties, (including mixed dishes)
11	**White meat**	Chicken, pork, turkey, (including mixed dishes)
12	**Fish and seafood**	Canned, fresh (including mixed dishes) and processed fish products e.g., fish balls
13	**Processed meat**	Corned beef (canned), corned silverside, smoked chicken, smoked hock and salami, ham, sausages, frankfurters, bacon, chorizo, luncheon meat
14	**Eggs**	Whole eggs (boiled, poached, fried, plain omelette) including egg mixed dishes (Quiche, frittata, omelette with filling)
15	**Legumes and meat alternatives**	Baked beans, black beans, dahl, canned or dried legumes, hummus, and legume based vegetarian meals and products (including meat alternatives and soy products edamame beans tofu, tempeh)

16	**Nuts and seeds**	Peanut butter and peanuts, Brazil nuts, walnuts, almond, cashew, pistachio, chia, linseed, pumpkin, sesame
17	**Animal fat**	Cream, sour cream, reduced cream, butter, lard, dripping, ghee
18	**Plant based fat**	Avocado (whole fruit), canola, sunflower, olive, vegetable oil, cooking spray, oil-based salad dressings (French/Italian), all margarines, coconut fats and products (fresh, oil, cream, milk)
19	**Savoury sauces and condiments**	Curry pastes, herb and spices, vinegar, gravy, sauces (tomato, barbeque, sweet chilli, mint, soy), mustard, chutney, miso, tomato paste, savoury spreads (vegemite, marmite) oil-based condiments (olives), pesto; creamy readymade meal-based sauces, dips, mayonnaise, aioli, tartare sauce, white sauce, cheese sauce; all soups (instant, canned, packet) and stocks
20	**Cakes and biscuits**	Cakes, biscuits, slices, loaves, muffins, doughnuts, sweet pies, pastries, tarts
21	**Puddings and desserts**	Puddings and desserts (semolina, pavlova, sticky date, fruit pies and crumbles), ice cream, custard, milk based puddings, jelly, ice blocks
22	**Sweet snacks and sugar**	Fruit and nut mixes, bliss balls, chocolate, lollies, muesli bars; all sugar added to food and drink including sweet spreads (Jam, honey, marmalade, syrup (maple, golden), Nutella, chocolate peanut butter, chocolate butter)
23	**Savoury snacks**	Popcorn, potato crisps, corn chips, twisties, bujha mix
24	**Crumbed and deep fried**	Hot chips/fries, hash browns, and packaged home baked chips, wontons, paraoa bread, schnitzel, nuggets, crumbed fish
25	**Fast-food (pizza & burgers)**	Fast-food burgers, burgers, pizzas, pies, dumplings, curries, noodle-based dishes, Nandos chicken, egg fu yong
26	**Fast food (sandwiches & sushi)**	Fast food: Salads, sandwiches, wraps, sushi, vegetable-based stir fry
27	**Sweetened beverages**	Fruit and/or vegetable juice and smoothies; soft drinks, cordials, flavoured water, sports drinks, soft drinks, fruit drinks, iced tea, energy drinks; All exclusively artificially sweetened beverages
28	**Tea, coffee, water**	Tea: Black, green, herbal, chai, kombucha; Coffee: instant, brewed, espresso, pre-mixed sachet, filter, cold brew. Water: Water (unflavoured, soda, tap)
29	**Alcoholic drinks**	Wine (standard and low alcohol) Beer (standard and low alcohol) Cider, spirits, RTDs, sherry, port, liqueurs, sake.

Supplementary Table S2. Associations between dietary fibre intake, body composition and metabolic markers for Pacific and NZ European women

Variable	β (95% CI) [a] n=284	p value	β (95% CI) [b e] n=284	p value	β (95% CI) [c] n=284	p value	β (95% CI) [d e] n=265	p value
**Body composition**								
Weight (kg)	-0.59 [-0.97, -0.20]	p=0.003	-0.59 [-0.97, -0.20]	p=0.003	-1.10 [-1.53, -0.66]	p<0.001	-1.03 [-1.45, -0.60]	p<0.001
BMI (kg/m2)	-0.25 [-0.38, -0.12]	p<0.001	-0.25 [-0.38, -0.12]	p<0.001	-0.38 [-0.53, -0.24]	p<0.001	-0.37 [-0.51, -0.23]	p<0.001
Total body fat %	-0.34 [-0.48, -0.21]	p<0.001	-0.34 [-0.48, -0.21]	p<0.001	-0.47 [-0.62, -0.31]	p<0.001	-0.44 [-0.60, -0.29]	p<0.001
Visceral fat %	-0.47 [-0.66, -0.29]	p<0.001	-0.47 [-0.66, -0.29]	p<0.001	-0.61 [-0.82, -0.40]	p<0.001	-0.58 [-0.80, -0.37]	p<0.001
**Blood pressure**[f]								
Systolic (mmHg)	-0.21 [-0.46, 0.03]	p=0.086	-0.09 [-0.33, 0.15]	p=0.476	-0.39 [-0.67, -0.11]	p=0.006	-0.23 [-0.51, 0.05]	p=0.108
Diastolic (mmHg)	-0.20 [-0.40, -0.002]	p=0.048	-0.03 [-0.22, 0.15]	p=0.726	-0.30 [-0.53, -0.07]	p=0.010	-0.06 [-0.27, 0.16]	p=0.605
**Metabolic markers**[f]								
TC (mmol/L)	-0.03 [-0.05, -0.01]	p=0.002	-0.03 [-0.04, -0.01]	p=0.006	-0.04 [-0.06, -0.02]	p=0.0004	-0.04 [-0.06, -0.01]	p=0.001
HDL-C (mmol/L)	**0.01 [0.002, 0.02]**	**p=0.018**	0.004 [-0.003, 0.01]	p=0.300	0.01 [-0.001, 0.02]	p=0.085	-0.001 [-0.01, 0.01]	p=0.743
LDL-C (mmol/L)	**-0.03 [-0.05, -0.02]**	**p=0.001**	**-0.03 [-0.05, -0.01]**	**p=0.002**	**-0.04 [-0.06, -0.02]**	**p=0.0003**	**-0.03 [-0.05, -0.01]**	**p=0.003**
TGS (mmol/L)	-0.01 [-0.02, 0.002]	p=0.112	-0.0005 [-0.01, 0.01]	p=0.923	**-0.02 [-0.03, -0.004]**	**p=0.009**	-0.01 [-0.02, 0.01]	p=0.368
HbA1c (mmol/L)[g]	-0.001 [-0.07, 0.04]	p=0.523	0.001 [-0.05, 0.06]	p=0.759	-0.04 [-0.10, 0.02]	p=0.229	-0.002 [-0.06, 0.06]	p=0.946
Fasting Glucose (mmol/L)	**-0.02 [-0.02, -0.01]**	**p=0.001**	**-0.01 [-0.02, -0.002]**	**p=0.019**	**-0.02 [-0.03, -0.01]**	**p=0.002**	-0.01 [-0.02, 0.001]	p=0.070
Fasting Insulin (uU/mL)[h]	**0.98 [0.97, 0.99]**	**p=0.001**	0.99 [0.98, 1.00]	p=0.146	**0.97 [0.96, 0.99]**	**p=0.0003**	-0.01 [-0.02, 0.004]	p=0.186

[a]Models adjusted for ethnicity, age, NZDep2013, and energy intake
[b]Models adjusted for ethnicity, age, NZDep2013, and energy intake,
[c]Models adjusted for ethnicity, age, NZDep2013, energy, protein, total fat, and carbohydrate intake
[d]Models adjusted for ethnicity, age, NZDep2013, energy, protein, total fat, and carbohydrate intake and physical activity (minutes per day spent in moderate to vigorous physical activity),
[e]blood pressure and metabolic markers further adjusted for body fat% group
Total n=284: NZ European n=160, Pacific n=124; [f]Pacific woman (n=1), and [g]Pacific women (n=3) not included in analyses due to missing data.
[h]data has been log transformed (ln)
Regression coefficients represent the change in the outcome per 1 g of change in dietary fibre intake

Supplementary Table S3. Associations between dietary fibre intake, body composition and metabolic markers for Pacific women

Variable	β (95% CI) [a] n=124	p value	β (95% CI) [b e] n=124	p value	β (95% CI) [c] n=124	p value	β (95% CI) [d e] n=108	p value
**Body composition**								
Weight (kg)	-0.54 [-1.32, 0.24]	p=0.171	-0.54 [-1.32, 0.24]	p=0.171	**-1.61 [-2.59, -0.63]**	**p=0.014**	**-1.37 [-2.37, -0.37]**	**p=0.008**
BMI (kg/m^2)	-0.24 [-0.50, 0.21]	p=0.071	-0.24 [-0.50, 0.21]	p=0.071	**-0.53 [-0.86, -0.21]**	**p=0.017**	**-0.50 [-0.84, -0.16]**	**p=0.005**
Total body fat %	**-0.24 [-0.46, -0.01]**	**p=0.038**	**-0.24 [-0.46, -0.01]**	**p=0.038**	**-0.48 [-0.77, -0.19]**	**p=0.016**	**-0.42 [-0.71, -0.13]**	**p=0.006**
Visceral fat %	**-0.32 [-0.64, -0.01]**	**p=0.045**	**-0.32 [-0.64, -0.01]**	**p=0.045**	**-0.59 [-1.01, -0.18]**	**p=0.006**	**-0.54 [-0.97, -0.11]**	**p=0.015**
**Blood pressure**								
Systolic (mmHg)	-0.21 [-0.60, 0.19]	p=0.300	-0.12 [-0.50, 0.25]	p=0.514	-0.45 [-0.98, 0.09]	p=0.099	-0.31 [-0.86, 0.23]	p=0.255
Diastolic (mmHg)	-0.11 [-0.45, 0.24]	p=0.549	0.003 [-0.31, 0.31]	p=0.984	-0.31 [-0.78, 0.16]	p=0.190	-0.05 [-0.49, 0.39]	p=0.816
**Metabolic markers** [f]								
TC (mmol/L)	0.001 [-0.03, 0.03]	p=0.949	0.001 [-0.03, 0.03]	p=0.942	-0.02 [-0.05, 0.02]	p=0.320	-0.03 [-0.07, 0.01]	p=0.135
HDL-C (mmol/L)	0.01 [-0.002, 0.02]	p=0.092	0.01 [-0.004, 0.02]	p=0.164	0.01 [-0.01, 0.02]	p=0.386	0.001 [-0.02, 0.02]	p=0.953
LDL-C (mmol/L)	-0.01 [-0.03, 0.02]	p=0.533	-0.01 [-0.03, 0.02]	p=0.568	-0.01 [-0.05, 0.02]	p=0.474	-0.02 [-0.06, 0.02]	p=0.319
TGS (mmol/L)	0.003 [-0.02, 0.02]	p=0.789	0.01 [-0.01, 0.03]	p=0.472	-0.02 [-0.05, 0.006]	p=0.128	-0.02 [-0.04, 0.01]	p=0.270
HbA1c (mmol/L) [g]	-0.04 [-0.16, 0.08]	p=0.517	-0.02 [-0.13, 0.10]	p=0.747	-0.09 [-0.26, 0.07]	p=0.260	-0.01 [-0.16, 0.15]	p=0.926
Fasting Glucose (mmol/L)	-0.02 [-0.04, 0.003]	p=0.104	-0.01 [-0.03, 0.01]	p=0.159	-0.02 [-0.04, 0.01]	p=0.221	-0.01 [-0.04, 0.02]	p=0.426
Fasting Insulin (uU/mL) [h]	**0.97 [0.94, 0.99]**	**p=0.012**	**0.97 [0.95, 1.00]**	**p=0.031**	**0.95 [0.92, 0.99]**	**p=0.008**	0.97 [0.96, 1.01]	p=0.099

[a]Models adjusted for age, NZDep2013, and energy intake
[b]Models adjusted for age, NZDep2013, and energy intake
[c]Models adjusted for age, NZDep2013, energy, protein, total fat, and carbohydrate intake
[d]Models adjusted for age, NZDep2013, energy, protein, total fat, and carbohydrate intake and physical activity (minutes per day spent in moderate to vigorous physical activity),
[e]blood pressure and metabolic markers further adjusted for body fat% group
[f]Pacific woman (n=1) and [g]Pacific women (n=3) not included in analyses due to missing data.
[h]data has been log transformed (ln)
Regression coefficients represent the change in the outcome per 1 g of change in dietary fibre intake

Supplementary Table S4. Associations between dietary fibre intake, body composition and metabolic markers for NZ European women

Variable	β (95% CI) [a] n=160	p value	β (95% CI) [b e] n=160	p value	β (95% CI) [c] n=160	p value	β (95% CI) [d e] n=155	p value
**Body composition**								
Weight (kg)	**-0.65 [-1.10, -0.20]**	**p=0.005**	**-0.65 [-1.10, -0.20]**	**p=0.005**	**-1.06 [-1.53, -0.59]**	**p<0.001**	**-0.99 [-1.46, -0.52]**	**p<0.001**
BMI (kg/m²)	**-0.27 [-0.42, -0.12]**	**p<0.001**	**-0.27 [-0.42, -0.12]**	**p<0.001**	**-0.38 [-0.53, -0.22]**	**p<0.001**	**-0.36 [-0.51, -0.20]**	**p<0.001**
Total body fat %	**-0.39 [-0.58, -0.21]**	**p<0.001**	**-0.39 [-0.58, -0.21]**	**p<0.001**	**-0.48 [-0.68, -0.28]**	**p<0.001**	**-0.45 [-0.65, -0.25]**	**p<0.001**
Visceral fat %	**-0.55 [-0.79, -0.30]**	**p<0.001**	**-0.55 [-0.79, -0.30]**	**p<0.001**	**-0.64 [-0.90, -0.37]**	**p<0.001**	**-0.60 [-0.87, -0.34]**	**p<0.001**
**Blood pressure** [f]								
Systolic (mmHg)	-0.27 [-0.60, 0.06]	p=0.106	-0.11 [-0.44, 0.21]	p=0.489	**-0.39 [-0.74, -0.05]**	**p=0.027**	-0.21 [-0.56, 0.13]	p=0.224
Diastolic (mmHg)	**-0.30 [-0.56, -0.04]**	**p=0.022**	-0.10 [-0.33, 0.13]	p=0.398	**-0.35 [-0.63, -0.07]**	**p=0.016**	-0.10 [-0.36, 0.16]	p=0.454
**Metabolic markers**								
TC (mmol/L)	**-0.04 [-0.07, -0.02]**	**p=0.001**	**-0.04 [-0.07, -0.01]**	**p=0.003**	**-0.04 [-0.07, -0.01]**	**p=0.004**	**-0.04 [-0.06, -0.01]**	**p=0.018**
HDL-C (mmol/L)	0.01 [-0.002, 0.02]	p=0.104	0.001 [-0.01, 0.01]	p=0.878	0.01 [-0.001, 0.02]	p=0.077	-0.0003 [-0.01, 0.01]	p=0.952
LDL-C (mmol/L)	**-0.05 [-0.07, -0.02]**	**p=0.001**	**-0.04 [-0.06, -0.01]**	**p=0.003**	**-0.04 [-0.07, -0.02]**	**p=0.002**	**-0.03 [-0.06, -0.01]**	**p=0.018**
TGS (mmol/L)	**-0.01 [-0.02, -0.002]**	**p=0.020**	-0.005 [-0.02, 0.01]	p=0.381	-0.02 [-0.03, -0.002]	p=0.021	-0.003 [-0.01, 0.01]	p=0.595
HbA1c (mmol/L)	-0.01 [-0.07, 0.05]	p=0.719	0.01 [-0.06, 0.07]	p=0.821	-0.03 [-0.10, 0.04]	p=0.372	-0.01 [-0.08, 0.06]	p=0.798
Fasting Glucose (mmol/L)	**-0.01 [-0.02, -0.004]**	**p=0.007**	-0.01 [-0.02, 0.002]	p=0.122	**-0.02 [-0.03, -0.01]**	**p=0.004**	-0.01 [-0.02, 0.003]	p=0.133
Fasting Insulin (uU/mL) [g]	**0.98 [0.97, 1.00]**	**p=0.022**	1.00 [0.99, 1.01]	p=0.757	**0.98 [0.96, 0.99]**	**p=0.001**	0.99 [0.98, 1.01]	p=0.321

[a]Models adjusted for age, NZDep2013, and energy intake
[b]Models adjusted for age, NZDep2013, and energy intake
[c]Models adjusted for age, NZDep2013, energy, protein, total fat, and carbohydrate intake
[d]Models adjusted for age, NZDep2013, energy, protein, total fat, and carbohydrate intake and physical activity (minutes per day spent in moderate to vigorous physical activity)
[e]blood pressure and metabolic markers further adjusted for body fat% group
[f]NZ European women (n=2) not included in analyses due to missing data.
[g]data has been log transformed (ln)
Regression coefficients represent the change in the outcome per 1 g of change in dietary fibre intake
